# Prisoner’s Dilemma Game with Cooperation-Defection Dominance Strategies on Correlational Multilayer Networks

**DOI:** 10.3390/e24060822

**Published:** 2022-06-13

**Authors:** Qin Li, Guopeng Zhao, Minyu Feng

**Affiliations:** 1 School of Public Policy and Administration, Chongqing University, Chongqing 400044, China; 20200101023@cqu.edu.cn; 2 College of Artificial Intelligence, Southwest University, Chongqing 400715, China; gpzhao0514@gmail.com

**Keywords:** multilayer networks, cooperation-defection dominance, BA network, WS network

## Abstract

As multilayer networks are widely applied in modern society, numerous studies have shown the impact of a multilayer network structure and the network nature on the proportion of cooperators in the network. In this paper, we use Barabási–Albert scale-free networks (BA) and Watts and Strogatz networks (WS) to build a multilayer network structure, and we propose a new strategy-updating rule called “cooperation-defection dominance”, which can be likened to dominant and recessive traits in biogenetics. With the newly constructed multilayer network structure and the strategy-updating rules, based on the simulation results, we find that in the BA-BA network, the cooperation dominance strategy can make the networks with different *r*s show a cooperative trend, while the defection dominance strategy only has an obvious effect on the network cooperation with a larger *r*. When the BA network is connected to the WS network, we find that the effect of strategy on the proportion of cooperators in the network decreases, and the main influencing factor is the structure of the network. In the three-layer network, the cooperation dominance strategy has a greater impact on the BA network, and the proportion of the cooperators is enhanced more than under the natural evolution strategy, but the promotion effect is still smaller than that of the two-layer BA network because of the WS network. Under the defection dominance strategy, the WS layer appears different from the first two strategies, and we conclude through simulation that when the payoff parameter is at the middle level, its cooperator proportion will be suppressed, and we deduce that the proportion of cooperators and defectors, as well as the payoff, play an important role.

## 1. Introduction

In today’s society, abundant networks are more and more widely used, and different social structures can be abstracted into different networks. In some studies, researchers have abstracted community structures, biological systems, and neural networks into network structures, which greatly inspires related studies [1,2]. The mutual influence among different social structures may lead to different levels of individuals. Furthermore, the behaviors and the essential structures of society are also important factors that affect individual activities. The research of multilayer networks is a hot issue that has been focused on economics [3], sociology [4], psychology [5], and biology [6]. Through research on this topic, we can improve our understanding of human behavior [7], social systems [8], and the ecological environment [9].

How to understand the emergence of cooperation in a structured human society is difficult [10]. As it had been generally believed that the Nash equilibrium of the prisoner’s dilemma is the defection strategy [11], in 1992, Nowak and May first combined evolutionary games and spatial chaos to explain the existence of cooperators among the weak prisoner’s dilemma players in the population [12]. Thereafter, numerous studies spring up to show that the spatial structures enhance the cooperative behaviors among the individuals [5]. However, the research on the snowdrift games (SDG) by Hauert and Doebeli promoted scientists to reexamine the effect of spatial structures [13], finding that the spatial chaos often inhibits the evolution of cooperation in the SDGs. With the development of network science, the evolution of cooperation on stochastic graphs has been studied as an extension of Nowak and May’s work. At the end of the last century, Watts and Strogatz introduced the small-world networks to explain the small-world phenomenon (popularly known as six degrees of separation) in real social systems [14]. However, the small-world network model ignored the growth and preferential attachment characteristics in many social networks, which inspired Barabási and Albert’s work on scale-free networks [15]. Based on these two network models, abundant studies have built up models to simulate the real systems [16], providing well-grounded spatial structures for the study of human cooperation. In accordance with these network models, Santos and Pacheco first studied the evolutionary weak prisoner’s dilemmas and the snowdrift games on the scale-free networks to explore the causes of the emergence of a large number of human cooperative behaviors [17], finding that the scale-free structures provide a unifying framework for the cooperators. In addition, the cooperation density of small-world networks changes with the homogeneity and heterogeneity of the network [18,19]. It is worth mentioning that the random graphs usually help to form more cooperators than the lattices. Additionally, more and more social phenomena have been introduced to the study of human cooperation as the development of complex networks [20,21].

In the last decade, the mathematical concept of multilayer networks has emerged as the network science required [2,22]. However, such an approach to analyzing social networks is not new and can date back to the seventies in the last century. In 1974, Erving Goffman introduced the concept of multilayer networks along with the theory of frame analysis to study multilayer social networks [23]. With the progress of network science, this concept is of concern again to study the online communities [24], the internet [25], the citation networks [26], the trade networks [27], and other social interactions [28,29]. Meanwhile, the multilayer network models somehow provide a framework to study the human cooperation and social dynamics that is closer to reality than the single-layer network models [30,31], where numerous studies pay attention to some specific social phenomenon that may influence cooperation in the human population. Huang et al. studied the effects of external forcing on evolutionary games in complex networks [32], finding that the mechanism of external forcing on the evolutionary game is a strong promoter of cooperation even under a severe temptation condition. Kleineberg et al. studied the topological enslavement in evolutionary games on correlated multiplex networks, finding that if the multiplex is composed of many layers and degree correlations are strong [33], the topology of the system enslaves the dynamics, and the final outcome, cooperation or defection, becomes independent of the payoff parameters. Chen et al. studied the evolution of cooperation driven by collective interdependence on multilayer networks [34], finding that collective interdependence impacts interdependent network reciprocity significantly and highlights the importance of network reciprocity in enhancing the evolution of cooperation.

The conclusion we introduced above and some studies on interdependent network reciprocity extend the study of multilayer networks. Mechanisms including coupled evolutionary fitness [35,36], interconnectedness [37,38,39], biased imitation [40,41], information sharing [42,43], separation of interaction and learning networks [44,45], as well as biased resource allocation [46] have shown that the multilayer network structure facilitates the evolution of cooperation, which enriches the literature on interdependent network reciprocity in the evolutionary game theory. For the sake of probing how interdependent network reciprocity affects the evolution of cooperation, it is of paramount importance to construct interdependence between multilayer networks.

Through previous research on the single-layer network, we know that the structure of the network in which nodes are located plays an important part in the emergence of cooperation; a typical example is that scale-free networks can promote node cooperation. Nevertheless, the situation in real life is frequently more complicated, and the social structure level is more changeable as well. To more clearly view the influence of the structure of the network on the nodes, we research the relationship between the two-layer network and the three-layer network in this article. To verify the model and facilitate our study, we consider the social structure as a combination of the BA scale-free networks and the WS small-world networks. In addition to the abstract depiction of social structure, another key point of the paper is that we propose strategy-updating rules, which is called “cooperation-defection dominance”. The strategy reflects the reality of a society in which the behavior of leaders has a significant impact on their subordinate members. We abstract leaders as a dominant strategy, and to achieve the effect, we refer to the dominant character and the recessive character in biogenetics, which needs the nodes to comply with the dominant strategy in this simulation. In short, when one node in a group of coupled nodes takes the dominant strategy, then whatever strategy the other nodes in the group are currently taking, they will take the dominant strategy during the game.

In the following section, we obtain the simulation results of the prisoner’s dilemma game (PDG) based on the two-layer network and the three-layer network, and we have made a comparative analysis of them, which obtains the relationship and difference.

## 2. Model

### 2.1. The Payoff Matrix of the Prisoner’s Dilemma Game

The payoff matrix describes the payoff of a node in the network when playing against another node under the PDG. Initially, individuals with different strategies, i.e., cooperation and defection, are supposed to be distributed evenly in the network. For each interaction, the individuals play the PDG (either to be a cooperator (C) or defector (D)) while the individual can adopt different strategies when interacting with different neighbors. Two cooperators are anticipated to both receive a reward of *R*; while two defectors will obtain the punishment of *P*. However, if two players choose different strategies, the cooperator will bear the sucker’s payoff *S*; whereas the defector earns the highest payoff *T*, where T>R>P>S, 2R≥T+S.

To easily change the payoff matrix, we unify the parameters into *r*. Specifically, for the PDG, we rewrite the payoff matrix as follows

(1) $$\left(\begin{array}{cc} R & S\\ T & P \end{array}\right)=\left(\begin{array}{cc} 1 & -r\\ 1+r & 0 \end{array}\right)$$

where *r* > 0.

### 2.2. Update Rules

The Fermi–Dirac function is the statistical distribution function often used in games; thus, we use it to calculate the probability of nodes updating in this paper. The meaning of the Fermi function is that a node *x* randomly selects one of its neighbor nodes *y*, and determines the probability that node *x* adopts the strategy of node *y* in the next round according to the payoff between the node *x* and the node *y*. The individual *x* imitates *y*’s strategy with the probability given by the Fermi function denoted as:

(2) $$W=\frac{1}{1+\exp\left[\left(\pi_{x}-\pi_{y}\right)/\kappa \right]}$$

where the $\pi_{x}$ denotes a round of the cumulative payoff of node *x* in the network, the $\pi_{y}$ indicates a round of the cumulative payoff of the node *y* in the network, and the κ indicates the selection intensity in the process of updating. For a given value of κ, if the total payoff of *x* is less than *y*, *x* tends to learn the strategy of *y*. The greater the difference in payoff between the two nodes, the greater the probability. However, if the total payoff of *x* is greater than *y*, it can learn the strategy of *y* as well with a small probability. When κ→ 0, updating the strategy becomes easy. If the payoff of the node *x* is a little less than node *y*, node *x* will adopt the strategy of node *y*. If κ→∞, the update of the strategy has a little connection with the payoff of nodes, so the process is dominated by the random drift, and the strategy update is stochastic.

### 2.3. Cooperation–Defection Dominance Strategy

The multilayer network is widely used in our daily life, and we find that there are leaders who manage these multilayered structures to prevent confusion. To study this structure, we simplify the network to two and three layers and introduce the cooperation–defection dominance strategy. Next, we will describe the multilayer networks and the cooperation–defection dominance strategy.

To connect the multilayer network, we individually number the nodes in each layer. As the number of nodes in each layer is the same, there is a one-to-one correspondence between them, and these nodes with the same number form a group to influence each other.

In our model, each individual in a round of the game has its label to indicate whether it is a cooperator or a defector, and the label only changes in the next round according to the specifics of the current round of the game. When a node changes its strategy due to the updated rules, the nodes it plays with will treat it as the updated node, but when counting the percentage of cooperators, it is based on the label of the individuals in the current round. Three update strategies are introduced as follows.

- 1. Cooperation dominance strategy:

A group of nodes with the same number constitutes a set of coupled nodes, and under the cooperation dominance condition, when there is a node using the cooperation dominance strategy, the other nodes will change the current strategy and use the dominant strategy.

**Figure 1:**
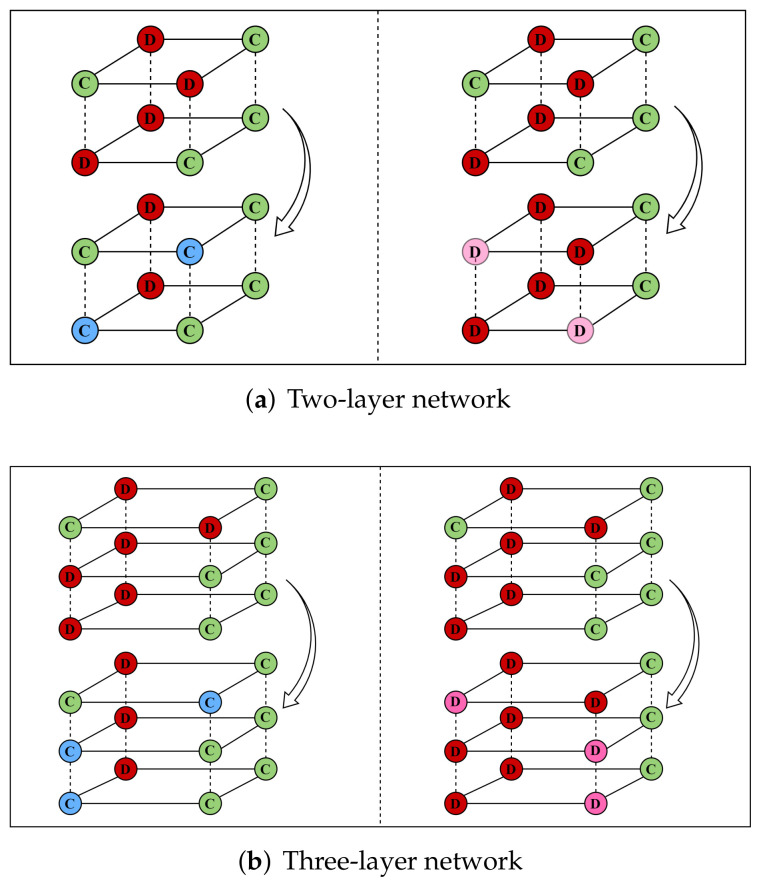
Multilayer network update demo diagram. Subgraphs (**a**,**b**) indicate the changes in the two-layer network and three-layer network under the cooperation-defection dominance strategy, respectively. The left side of the dotted line indicates the cooperation dominance strategy, and the right side indicates the defection dominance strategy. In the figure, cooperators are denoted by C, and defectors are denoted by D. The green nodes indicate the nodes that adopt the cooperation strategy, and the red nodes indicate the nodes that adopt the defection strategy. The blue nodes indicate the nodes that convert from adopting the defection strategy to adopting the cooperation strategy under the cooperation dominance strategy, and the pink nodes indicate the nodes that convert from adopting the cooperation strategy to adopting the defection strategy under the defection dominance strategy.

To better understand the new strategy, we present a simple example, as shown in Figure 1, the left side of the dashed lines in Figure 1a,b display the cooperation dominance strategy in two-layer and three-layer networks, respectively. We can see that regardless of the two-layer or three-layer structure of a network, the nodes of each layer correspond to each other one by one, the strategy of cooperation nodes will remain the same, and the rest nodes in the same group will follow the cooperation strategy. Specifically, the red node (defector), which is connected to the green node (cooperator) in the diagram, becomes the blue node (the cooperator converted from the defector node). In this round of the game, the blue node will play with other nodes as a cooperator and then judge what strategy the node will adopt in the next round according to the gain of the game.

- 2. Defection dominance strategy:

Similarly, when a node in the coupled nodes adopts the defection strategy under the defection dominance condition, the rest of the nodes in the group will only go to the defection strategy. As shown in Figure 1, the right side of the dashed lines in Figure 1a,b display the case of the defection dominant strategy. Only when the connected nodes at each level are all the cooperators, their strategy will remain the same. In other situations, as long as there are nodes with the defection strategy, the remaining nodes in this group will learn the defection strategy. Specifically, the green node (cooperator) connected to the red node (defector) in the diagram becomes the pink node (the defector node converted from the cooperator node). In this round of the game, the pink node will play as a betrayer with other nodes. Similarly, the next round of the node’s strategy is judged based on the gain of the game.

- 3. Natural evolution strategy:

To highlight the influence of the new strategy in the simulation section, we also set up a natural evolution strategy, which means that the nodes in each layer of the network are not affected by other layers, and only play games with the nodes in the same layer.

## 3. Results

The simulations are divided into three subsections. Concretely, we investigate the impact of new strategies on two-layer networks in the first subsection, study the influence of new strategies on three-layer networks and compare the simulation results with two-layer networks in the second subsection, and explain the abnormal result we found in three-layer simulations in the last subsection.

For simplicity, the number of network nodes is 500 in this manuscript, where the average degree of BA scale-free network is four. The random reconnection probability and the initial number of adjacent edges of the WS small-world networks are 0.5 and 4, respectively. In this simulation, the BA network with 500 nodes is chosen to conform to the general social collective structure.

The following simulations are carried out on the BA network and the WS network with 500 nodes, and the value of κ in Equation (2) is set to 0.1. The BA network and the WS network are constructed in *Python*. At the initial moment, the ratio of cooperators in the BA network is set to 0.5, while the WS network is set to 0.3, which is different from the BA network. In addition, we utilize three different payoff parameters *r*s (0.2, 0.5, and 0.8) to explore the evolution of network cooperation behavior under different payoff matrices.

### 3.1. Two-Layer Network Analysis

**Figure 2:**
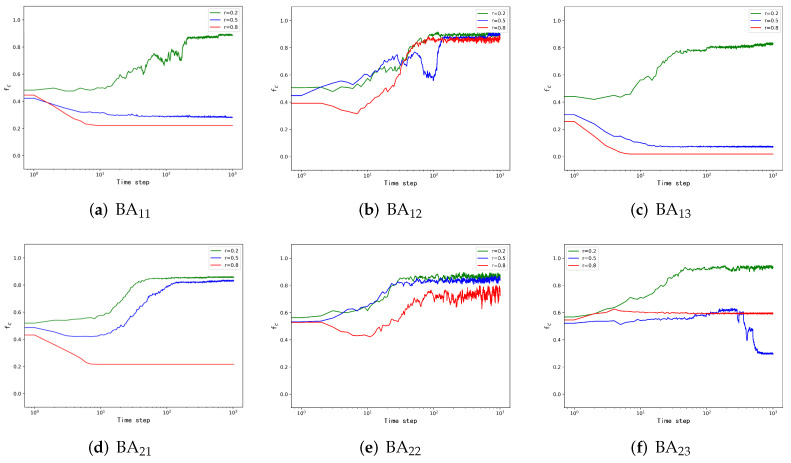
Evolution of the cooperation frequency on the BA-BA network as time progresses. In this simulation, the number of nodes in each layer of the network is 500, and the ratio of initial cooperators in the two BA networks is the same, and both are 0.5. We set the simulation steps (Time step = 1000), and the three different colored curves in the figure represent the corresponding payoff matrices under different payoff matrices. The *i* in BA${}_{ij}$ denotes the position of the layer in the multilayer network, and *j* represents the strategy-updating rule adopted (see the introduction to the strategy in Model).

**Figure 3:**
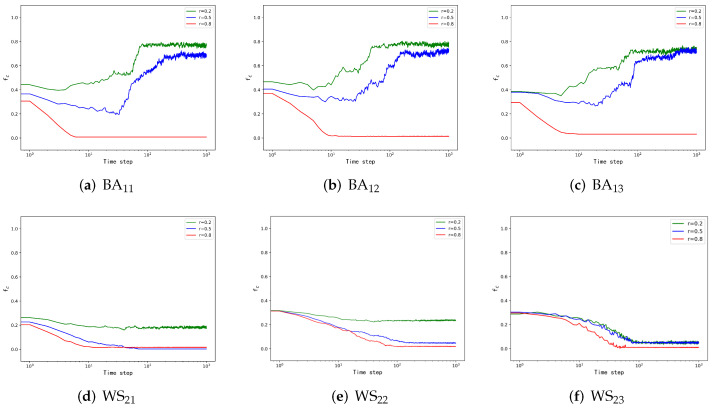
BA–WS network cooperators ratio change diagram. In this experiment, the number of individuals in each layer of the network is N = 500, and the initial partner ratios of the first layer of the BA network and the second layer of the WS small-world network are *f*${}_{1}$ = 0.5 and *f*${}_{2}$ = 0.3, respectively. The number of experimental steps is Time step = 1000, and the three different colored curves in the figure represent the corresponding payoff matrices under different payoff matrices. The *i* in BA${}_{ij}$ denotes the position of the BA network in the multilayer network and *j* represents the strategy-updating rule adopted (see the introduction to the strategy in Model), and WS${}_{ij}$ is interpreted in the same way as BA${}_{ij}$.

To study the influence of node behavior by strategy-updating rules and the two-layer network structure, we set up the BA-BA network (Figure 2) and the BA–WS network (Figure 3), respectively. Through the simulations, we obtain the differences in the proportion of cooperators at each layer when nodes take the cooperation dominance strategy, defection dominance strategy, and natural evolution strategy.

#### 3.1.1. BA-BA Network

It has been proposed that the BA network structure has a promoting effect on cooperation. Based on this, we explore whether the two-layer BA network still has such a promoting effect under our strategy-updating rules in this subsection. Hereby, we carry out three strategy-updating rules (introduced in Model) and obtain conclusions by comparing the simulation results of the same payoff parameter *r*.

In the BA-BA network simulation, we set the size of both networks to be the same and divide the simulations into three parts with three strategy-updating rules. Each simulation performs 1000 steps. As the proportion of cooperators stays stable, we gain the simulation results, as shown in Figure 2. The upper and lower layers correspond to the first and second layers of the network, respectively. Figure 2a,d demonstrates the proportion of cooperators in the two layers corresponding to different payoff parameters *r*s during the natural evolution. By analyzing the simulation results, we can obtain that the cooperation behavior is promoted in the two-layer BA network when *r* = 0.2. However, if *r* rises to 0.8, the proportion of cooperators will drop rapidly and stabilize at an extremely low level, as shown in Figure 2a. In Figure 2d, the proportion of cooperators in the network is elevated when *r* = 0.5, which is different from Figure 2a. The reason for this is that the network randomly assigns cooperators in different ways, so the proportion of cooperators at stabilization will be different. In addition, the density of cooperators will eventually decrease as time increases when *r* = 0.8, which is the same as on the first layer BA network.

Although the proportion of cooperators is mostly decided by the structure of the network in the natural evolution, the strategy-updating rule is still significant for the stable proportion of cooperators. Figure 2b,e displays the proportion of cooperators under the cooperation dominance strategies. Through simulation results, we find that nodes in the network rapidly evolve towards cooperation, and the proportion of cooperators in the network is higher than the initial value in all cases of *r*s. When adopting the cooperation dominant strategy, no matter how the initial cooperation nodes are allocated, the probability of a pair of coupled nodes showing a cooperative state is much greater than that of both showing a defective state, so as *r* takes different values, cooperation will be promoted. Finally, compared with the natural evolution strategy-updating rule, although this rule cannot make the proportion of cooperators corresponding to each *r* larger than the natural evolution, it can make the networks with different *r*s show a cooperative trend.

Similarly, to further explore the function of the strategy-updating rules and observe the impact of the defection dominance strategy, we analyze Figure 2c,f, which display the proportion of first-layer and second-layer cooperators. In this case, if *r* is small, e.g., 0.2, the proportion of cooperators will be promoted, but when *r* is larger, e.g., 0.5 or 0.8, the proportion of cooperators will be inhibited. By comparing the proportion of cooperators under the defection dominance strategy with the other two strategy-updating rules, we suggest that the defection dominance strategy has little effect on the cooperation of the network with small *r* (*r* = 0.2) but has a significant inhibitory effect on the cooperation of the network with larger *r* (*r* = 0.5 and 0.8).

#### 3.1.2. BA–WS Network

Following the BA-BA network simulations, to reflect the influence of different network structures, we introduced the WS network, which inhibits the trend of nodes’ cooperation. In this subsection, we connect the BA network with the WS network and explore the effect of the interconnection of two different networks through the simulation results.

In the simulation, Figure 3a,d displays the BA network and the WS network, respectively, with the natural evolution strategies. As seen in Figure 3a, corresponding to three different *r*s, the proportion of cooperators shows an increasing trend when *r* is small and a decreasing trend when *r* is large, which is the same as in Figure 2a. In Figure 3d, the proportion of cooperators shows a decreasing trend, while the maximum proportion of cooperators in Figure 3d is smaller than the minimum proportion of cooperators in Figure 2d, which is caused by the structure of the WS network inhibiting cooperation.

Through the previous research, the WS network has a strong inhibitory effect on the cooperation of nodes. To explore the influence of the WS network on cooperators under the cooperation dominance strategy, we present Figure 3b,e to display the simulation results. Comparing Figure 2b and Figure 3b, the proportion of cooperators in the BA network structure is lower than in Figure 2b, while the tendency of cooperation in the network is also suppressed when *r* = 0.8, indicating that the structure of the WS network inhibits the promotion of cooperation in the BA network. Comparing Figure 3e with Figure 3d, we suggest that the proportion of cooperators under the cooperation dominance strategy was slightly higher than that of the natural evolution strategy, which shows that this strategy-updating rule has less impact on the WS network.

To investigate the role of the defection dominance strategy on BA and WS networks, we plotted Figure 3c,f. We can learn from Figure 3c that in the case of defection dominance, the smaller *r* promotes cooperation. When *r* is 0.8, the proportion of cooperators shows a suppressed state, which is the same as Figure 3a,b. As shown in Figure 3f, the trend of cooperation in the WS network decreases rapidly for all three different *r*s due to the strategy-updating rules and network structure. When *r* is 0.2 and 0.5, their stabilized cooperator proportions are approximately the same, and when *r* = 0.8, their cooperator proportions drop rapidly to very low levels.

Through simulation, we can see that the cooperation dominance strategy plays a small role in the cooperation evolution of the network in the BA–WS network, while the defection dominant strategy can inhibit the cooperation in the WS network to a larger extent.

### 3.2. Three-Layer Network Analysis

In the BA–WS network simulation, we analyzed that the WS network inhibits the tendency of cooperation for nodes that adopt different *r*s. Based on the obtained results, we next explore how the three-layer network structure affects cooperation under the new strategy. Combined with the simulation of the two-layer network, we further explore the role of the BA–WS–BA network structure on cooperation. The reason for choosing another BA network is to compare with the simulation results of the BA–WS network.

**Figure 4:**
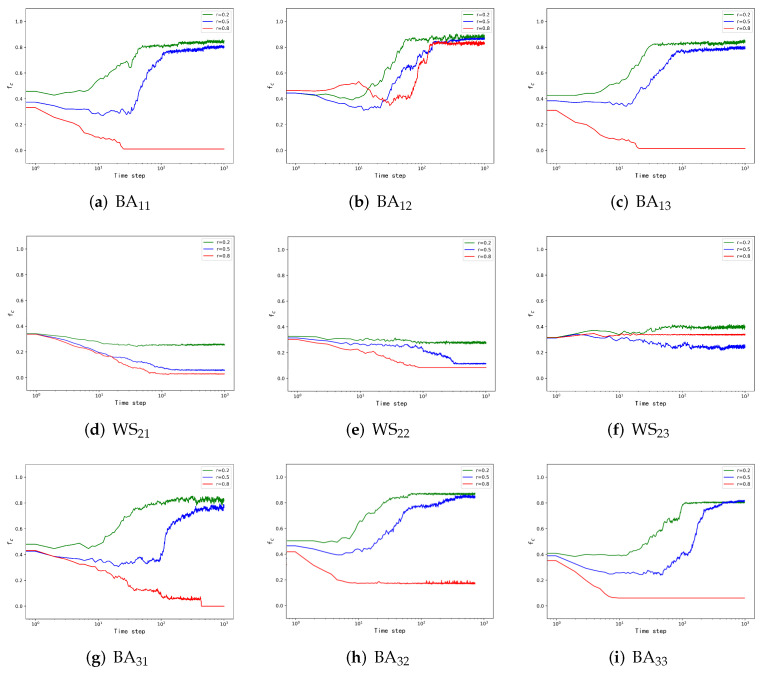
The proportion of three-layer network cooperators. In this simulation, the number of individuals in each layer of the network is N = 500. The initial partner ratios of the first-layer BA network, the second-layer WS small-world network, and the third-layer BA network are *f*${}_{1}$ = 0.5, *f*${}_{2}$ = 0.3, *f${}_{3}$* = 0.5, and the number of experimental steps is Time step = 1000. The three different color curves in the figure represent the corresponding payoff matrices under different payoff matrices. The *i* in BA${}_{ij}$ denotes the position of the BA network in the multilayer network, and *j* represents the strategy-updating rule adopted (see the introduction to the strategy in Model), and WS${}_{ij}$ is interpreted in the same way as BA ${}_{ij}$.

Figure 4a,d,g demonstrates the simulation results of different layers under the natural evolution strategy. Comparing Figure 4a,d,g when the network is a BA network, the corresponding cooperator proportions show an increasing trend when *r* is taken as 0.2 and 0.5, and when *r* is taken as 0.8, the cooperation income decreases, and the proportion of cooperators is less than the initial value when it is stable. When it is a WS network, the structure makes the cooperative behavior of nodes inhibited, and the proportion of cooperators decreases with the increase of time step. Moreover, when the layer is in a steady state, the proportion of cooperators decreases sequentially with an increasing *r*. This conclusion is consistent with the one obtained for the two-layer network.

Figure 4b,e,h explains the changes in the network of each layer under the condition of cooperation dominance strategy. As shown in Figure 4b, the nodes on the BA network have a strong tendency to cooperate, the network quickly reaches the state of high cooperation level, and the inhibitory effect of large *r* is not obvious. Comparing Figure 4d and Figure 4e we can find that the cooperative behavior of nodes in this layer is still inhibited under the influence of the WS network structure, and the cooperation dominance strategy only reduces the effect of inhibition. Comparing Figure 4b and Figure 4h, we find that the proportion of cooperators in Figure 4h is smaller than the initial value when *r* = 0.8, which is different from Figure 4b, and the reason is the random initialization of cooperators. However, comparing Figure 4g,h, we find that the cooperation dominance strategy results in a higher proportion of cooperators at different *r*. This phenomenon suggests that the cooperation dominance strategy has a role in facilitating network cooperation but less so under the influence of WS networks.

Figure 4c,f,i displays the results with the defection dominance strategy. As shown in Figure 4c,i, the proportion of cooperators is lower than that in the cooperation dominance strategy. However, as shown in Figure 4f, influenced by the structure and strategy-updating rules, a larger *r* and a smaller *r*, e.g., 0.8 and 0.2 in WS networks, appear to promote cooperation, while *r* at intermediate levels inhibit cooperation. Comparing three different *r*s, when *r* is less than 0.5, the proportion of cooperators will decrease with the increase of *r*. However, we observe that when *r* = 0.8, the proportion of collaborators is larger than when *r* = 0.5. We suggest that when *r* is large, there exist many defection nodes in the network and exist special cases in which the nodes in a group are all cooperators (introduced in Model), and when most of the nodes take the defection strategy, they probably do not gain as much payoff as the cooperators connected to it in the next game round. Therefore, the nodes will choose the cooperation strategy in the next round, and the proportion of cooperators is higher than the other smaller *r* groups. We will further investigate this phenomenon through a simulation.

### 3.3. Special Case Analysis in WS Network

**Figure 5:**
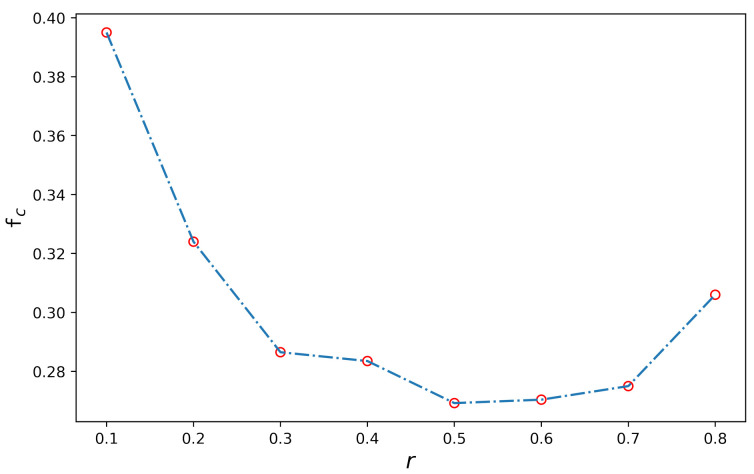
Schematic diagram of changes in the proportion of cooperators in the WS network. The figure shows the change in the proportion of cooperators in the WS network with *r* when the node is in the defection dominance condition. The abscissa represents the time step, the ordinate *r* is the parameter in the payoff matrix, and the ordinate represents the proportion of cooperators.

By analyzing the three-layer network, we obtained the effect of the three-layer structure on the tendency of nodes to cooperate and the effect of different dominant strategies on the proportion of cooperators in each layer. However, as mentioned in the three-layer network simulation, when *r* = 0.8, the WS network structure will lead to an increase in the proportion of cooperators in this layer. To explain this phenomenon, we improved the three-layer network simulation and obtained the proportion of cooperators corresponding to different *r* in the WS network separately, and the simulation results are shown in Figure 5.

In Figure 5, we can obtain the proportion of cooperators corresponding to the eight different *r*. When *r* = 0.1, the proportion of cooperators is the highest, which is about 40%. When *r* increases to 0.5, the proportion of cooperators stabilizes at 28%, at the lowest level of the simulation. However, with the increase of *r*, the proportion of cooperators will slowly increase. When *r* increases to 0.8, the proportion of collaborators is roughly 31%, which is bigger than the result when *r* = 0.5. Through the analysis, we found that the proportion of cooperators shows a tendency to decrease and then increase as *r* increases and achieves the minimum value at *r* = 0.5. Through this simulation conclusion, we can explain that the proportion of cooperators will be greater than in other cases when *r* = 0.8 in the WS network.

## 4. Discussion

In this paper, we introduce a new strategy-updating rule called “cooperation–defection dominance” to the evolutionary game model in the multilayer networks. The simulation results show that when the structure of the network promotes the cooperative behavior of the nodes in the network, most of the nodes in this layer will adopt the cooperative strategy regardless of which strategy we adopt. Under the cooperation dominance strategy, the proportion of cooperators is higher than in other strategies, but if the network structure of this layer inhibits the cooperation of nodes, the cooperation tendency of nodes will be inhibited no matter which strategy-updating rules we adopt. Meanwhile, different strategies can only partially affect the proportion of cooperators as the network structure is determined. In addition, the ability of the BA network structure to facilitate node cooperation is inhibited if the BA network connects to the WS network. With the simulation results, we also find that the proportion of cooperators under the defection dominance strategy is larger than the proportion of the other two strategies in the WS network. In most simulations, small *r* values correspond to large cooperator proportions; however, when we choose the WS network for simulations under defection domination, we find that the relationship between cooperator proportions and *r* is not monotonic in the WS layer network. In WS networks, *r* is the middle value that inhibits the cooperation of nodes by our simulation results.

Through the simulations, we learn the regularities of the multilayer network games with the strategies we set, but we only introduced the one-to-one correspondence in networks and only analyze the relationship between BA networks and WS networks. More complex network structures, better update strategies, and a variety of different game models can be employed to study conclusions related to multilayer networks in the future.

## Data Availability

Not applicable.

## Abbreviations

The following abbreviations are used in this manuscript:

SDG	the snowdrift games
PDG	the prisoner’s dilemma games
BA network	the Barabási–Albert scale-free network
WS network	the Watts and Strogatz network

## References

[1] Boccaletti S., Latora V., Moreno Y., Chavez M., Hwang D.U. (2006). Complex networks: Structure and dynamics. Phys. Rep..

[2] Boccaletti S., Bianconi G., Criado R., Del Genio C.I., Gómez-Gardenes J., Romance M., Sendina-Nadal I., Wang Z., Zanin M. (2014). The structure and dynamics of multilayer networks. Phys. Rep..

[3] Chen X., Szolnoki A., Perc M. (2015). Competition and cooperation among different punishing strategies in the spatial public goods game. Phys. Rev. E.

[4] Stewart A.J., Plotkin J.B. (2014). Collapse of cooperation in evolving games. Proc. Natl. Acad. Sci. USA.

[5] Szabó G., Fath G. (2007). Evolutionary games on graphs. Phys. Rep..

[6] Altman E., Reiffers A., Menasche D.S., Datar M., Dhamal S., Touati C. (2019). Mining competition in a multi-cryptocurrency ecosystem at the network edge: A congestion game approach. ACM Sigmetrics Perform. Eval. Rev..

[7] Cardillo A., Gómez-Gardeñes J., Vilone D., Sánchez A. (2010). Co-evolution of strategies and update rules in the prisoner’s dilemma game on complex networks. New J. Phys..

[8] Wang Z., Perc M. (2010). Aspiring to the fittest and promotion of cooperation in the prisoner’s dilemma game. Phys. Rev. E.

[9] Perc M., Szolnoki A. (2010). Coevolutionary games—A mini review. BioSystems.

[10] Rand D.G., Nowak M.A. (2013). Human cooperation. Trends Cogn. Sci..

[11] Axelrod R. (1980). Effective choice in the prisoner’s dilemma. J. Confl. Resolut..

[12] Nowak M.A., May R.M. (1992). Evolutionary games and spatial chaos. Nature.

[13] Hauert C., Doebeli M. (2004). Spatial structure often inhibits the evolution of cooperation in the snowdrift game. Nature.

[14] Watts D.J., Strogatz S.H. (1998). Collective dynamics of ‘small-world’networks. Nature.

[15] Barabási A.L., Albert R. (1999). Emergence of scaling in random networks. Science.

[16] Albert R., Barabási A.L. (2002). Statistical mechanics of complex networks. Rev. Mod. Phys..

[17] Santos F.C., Pacheco J.M. (2005). Scale-free networks provide a unifying framework for the emergence of cooperation. Phys. Rev. Lett..

[18] Hauert C., Szabó G. (2005). Game theory and physics. Am. J. Phys..

[19] Santos F.C., Rodrigues J.F., Pacheco J.M. (2005). Epidemic spreading and cooperation dynamics on homogeneous small-world networks. Phys. Rev. E.

[20] Zeng Z., Li Y., Feng M. (2022). The spatial inheritance enhances cooperation in weak prisoner’s dilemmas with agents’ exponential lifespan. Phys. A Stat. Mech. Its Appl..

[21] Pi B., Zeng Z., Feng M., Kurths J. (2022). Evolutionary multigame with conformists and profiteers based on dynamic complex networks. Chaos Interdiscip. J. Nonlinear Sci..

[22] Kivelä M., Arenas A., Barthelemy M., Gleeson J.P., Moreno Y., Porter M.A. (2014). Multilayer networks. J. Complex Netw..

[23] Goffman E. (1974). Frame Analysis: An Essay on the Organization of Experience.

[24] Castronova E. (2008). Synthetic worlds. Synthetic Worlds.

[25] Barnett G.A., Park H.W., Jiang K., Tang C., Aguillo I.F. (2014). A multi-level network analysis of web-citations among the world’s universities. Scientometrics.

[26] Sun J., Tao D., Faloutsos C. Beyond streams and graphs: Dynamic tensor analysis. Proceedings of the 12th ACM SIGKDD International Conference on Knowledge Discovery and Data Mining.

[27] Alves L.G.A., Mangioni G., Rodrigues F.A., Panzarasa P., Moreno Y. (2018). Unfolding the complexity of the global value chain: Strength and entropy in the single-layer, multiplex, and multi-layer international trade networks. Entropy.

[28] Zignani M., Quadri C., Gaitto S., Rossi G.P. (2014). Exploiting all phone media? A multidimensional network analysis of phone users’ sociality. arXiv.

[29] Formichini M., Cimini G., Pugliese E., Gabrielli A. (2019). Influence of technological innovations on industrial production: A motif analysis on the multilayer network. Entropy.

[30] Wang Z., Wang L., Szolnoki A., Perc M. (2015). Evolutionary games on multilayer networks: A colloquium. Eur. Phys. J. B.

[31] Bernal Jaquez R., Alarcón Ramos L.A., Schaum A. (2020). Spreading Control in Two-Layer Multiplex Networks. Entropy.

[32] Huang K., Zhang Y., Li Y., Yang C., Wang Z. (2018). Effects of external forcing on evolutionary games in complex networks. Chaos Interdiscip. J. Nonlinear Sci..

[33] Kleineberg K.K., Helbing D. (2018). Topological enslavement in evolutionary games on correlated multiplex networks. New J. Phys..

[34] Chen W., Yang Z., Wu T. (2021). Evolution of cooperation driven by collective interdependence on multilayer networks. Appl. Math. Comput..

[35] Wang Z., Szolnoki A., Perc M. (2013). Optimal interdependence between networks for the evolution of cooperation. Sci. Rep..

[36] Wang Z., Szolnoki A., Perc M. (2014). Self-organization towards optimally interdependent networks by means of coevolution. New J. Phys..

[37] Gómez-Gardenes J., Gracia-Lázaro C., Floria L.M., Moreno Y. (2012). Evolutionary dynamics on interdependent populations. Phys. Rev. E.

[38] Li H., Dai Q., Cheng H., Yang J. (2010). Effects of inter-connections between two communities on cooperation in the spatial prisoner’s dilemma game. New J. Phys..

[39] Wang B., Chen X., Wang L. (2012). Probabilistic interconnection between interdependent networks promotes cooperation in the public goods game. J. Stat. Mech. Theory Exp..

[40] Wang B., Pei Z., Wang L. (2014). Evolutionary dynamics of cooperation on interdependent networks with the Prisoner’s Dilemma and Snowdrift Game. EPL (Europhys. Lett.).

[41] Santos M.D., Dorogovtsev S.N., Mendes J.F. (2014). Biased imitation in coupled evolutionary games in interdependent networks. Sci. Rep..

[42] Szolnoki A., Perc M. (2013). Information sharing promotes prosocial behaviour. New J. Phys..

[43] Xia C., Li X., Wang Z., Perc M. (2018). Doubly effects of information sharing on interdependent network reciprocity. New J. Phys..

[44] Ohtsuki H., Nowak M.A., Pacheco J.M. (2007). Breaking the symmetry between interaction and replacement in evolutionary dynamics on graphs. Phys. Rev. Lett..

[45] Ohtsuki H., Pacheco J.M., Nowak M.A. (2007). Evolutionary graph theory: Breaking the symmetry between interaction and replacement. J. Theor. Biol..

[46] Chen W., Wu T., Li Z., Wang L. (2017). Randomly biased investments and the evolution of public goods on interdependent networks. Phys. A Stat. Mech. Its Appl..

